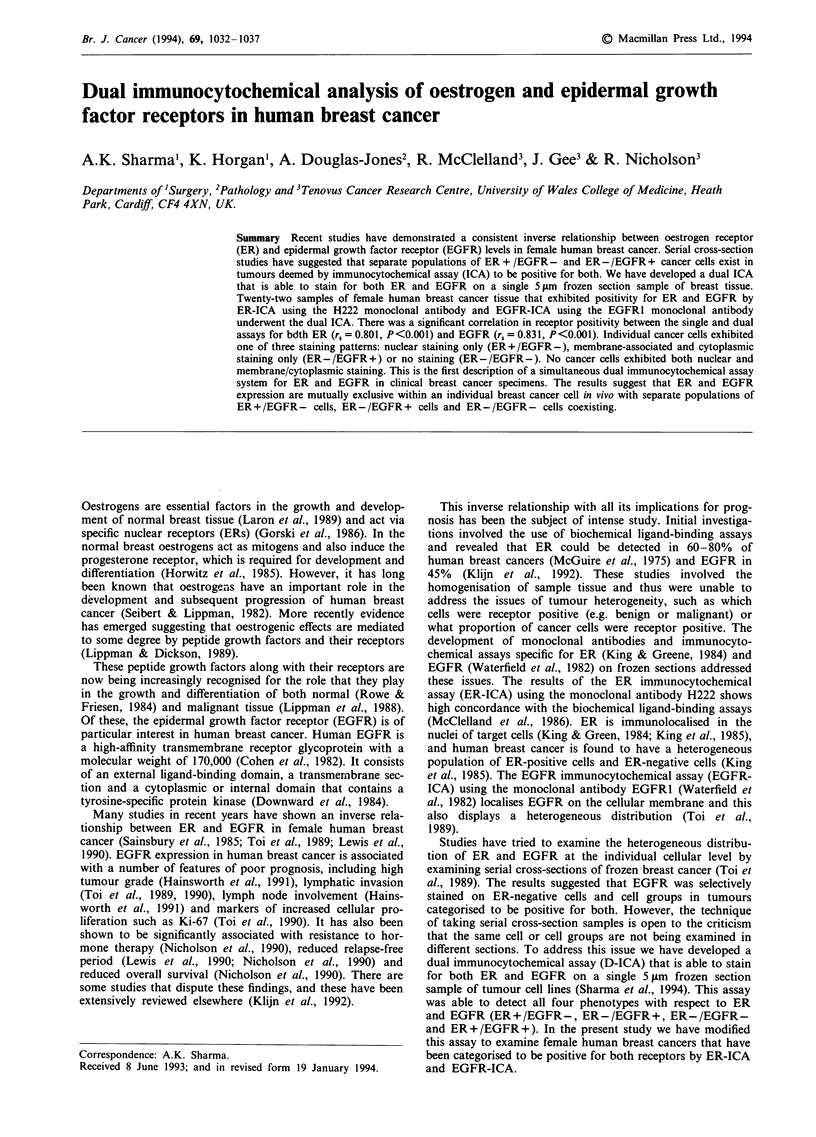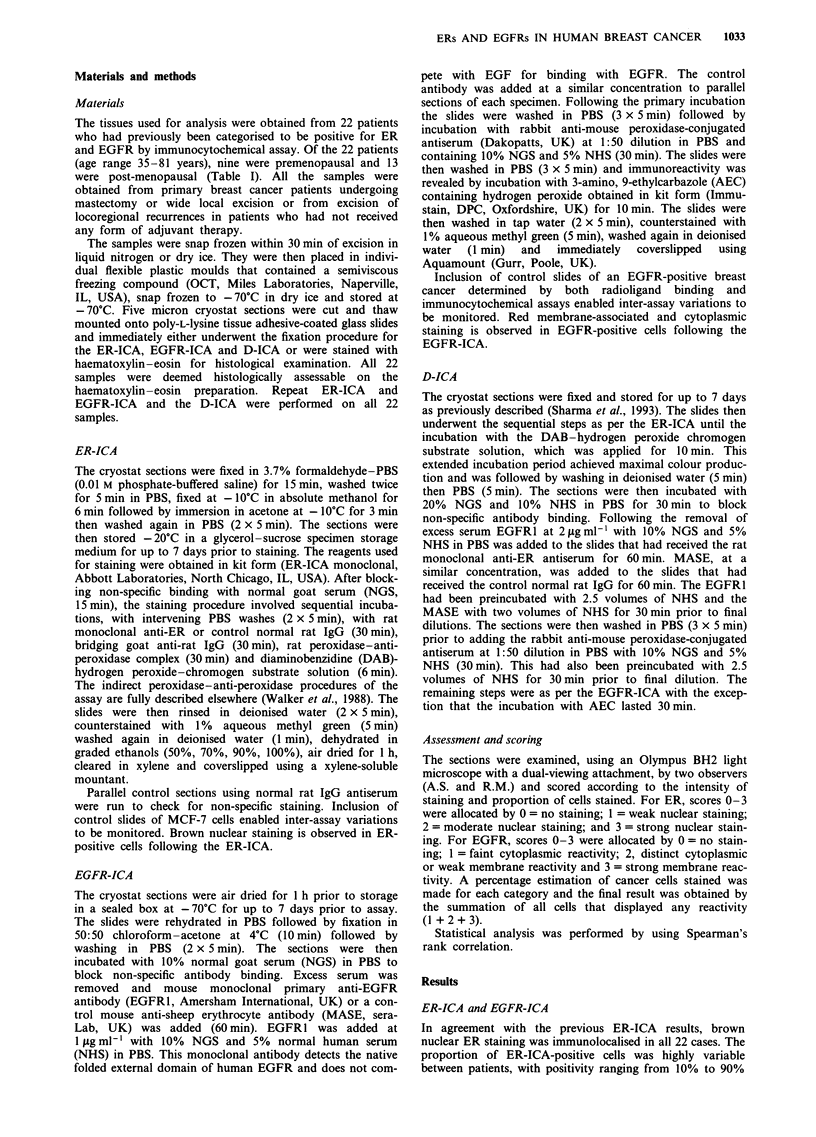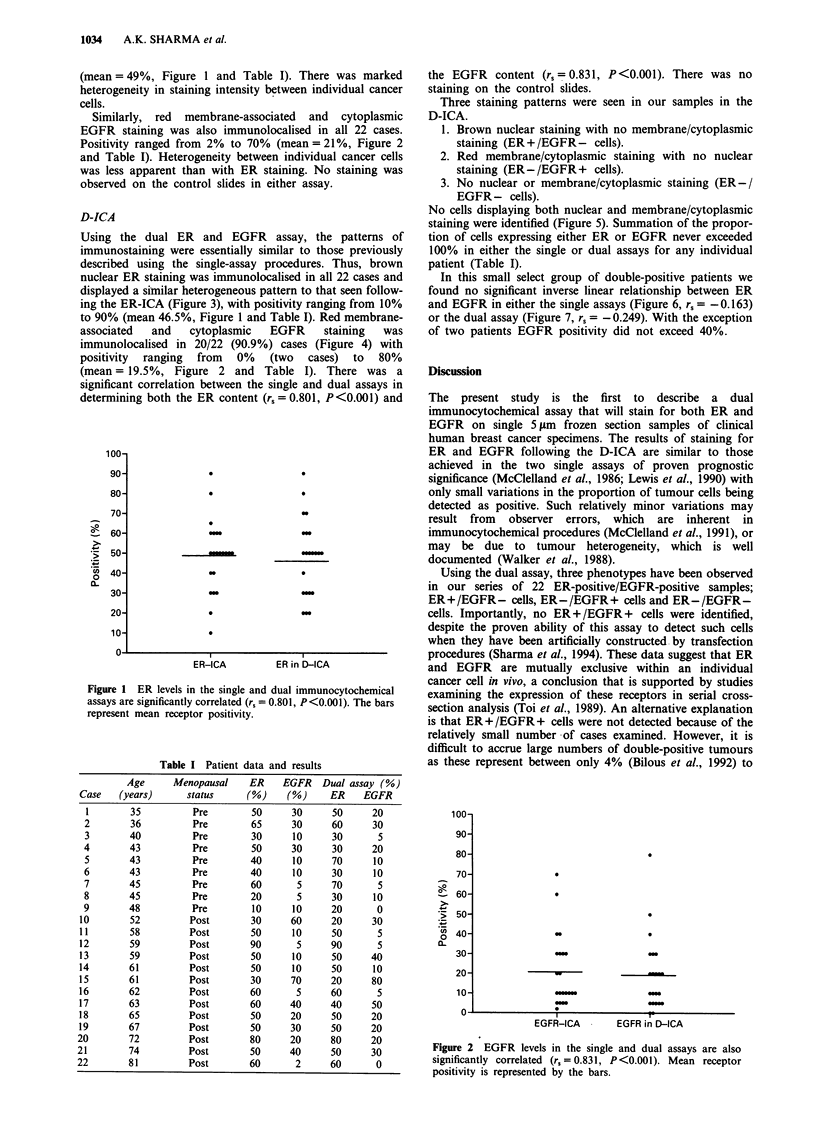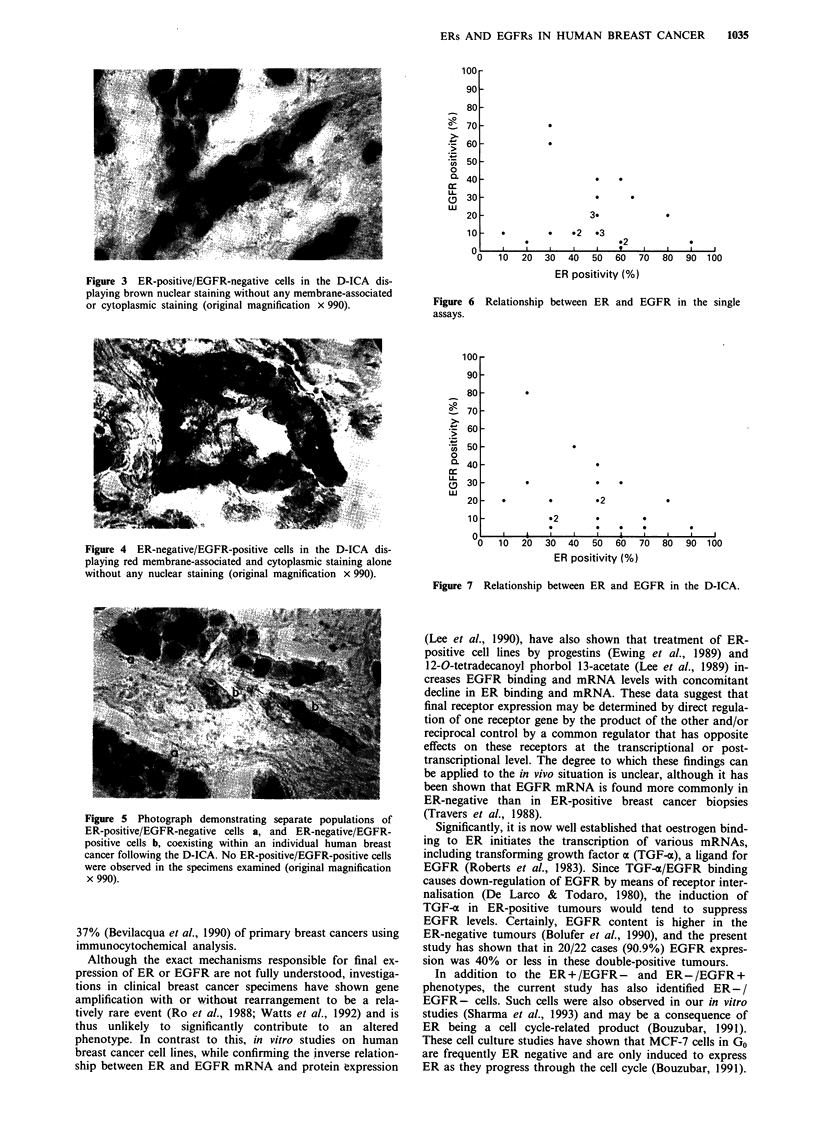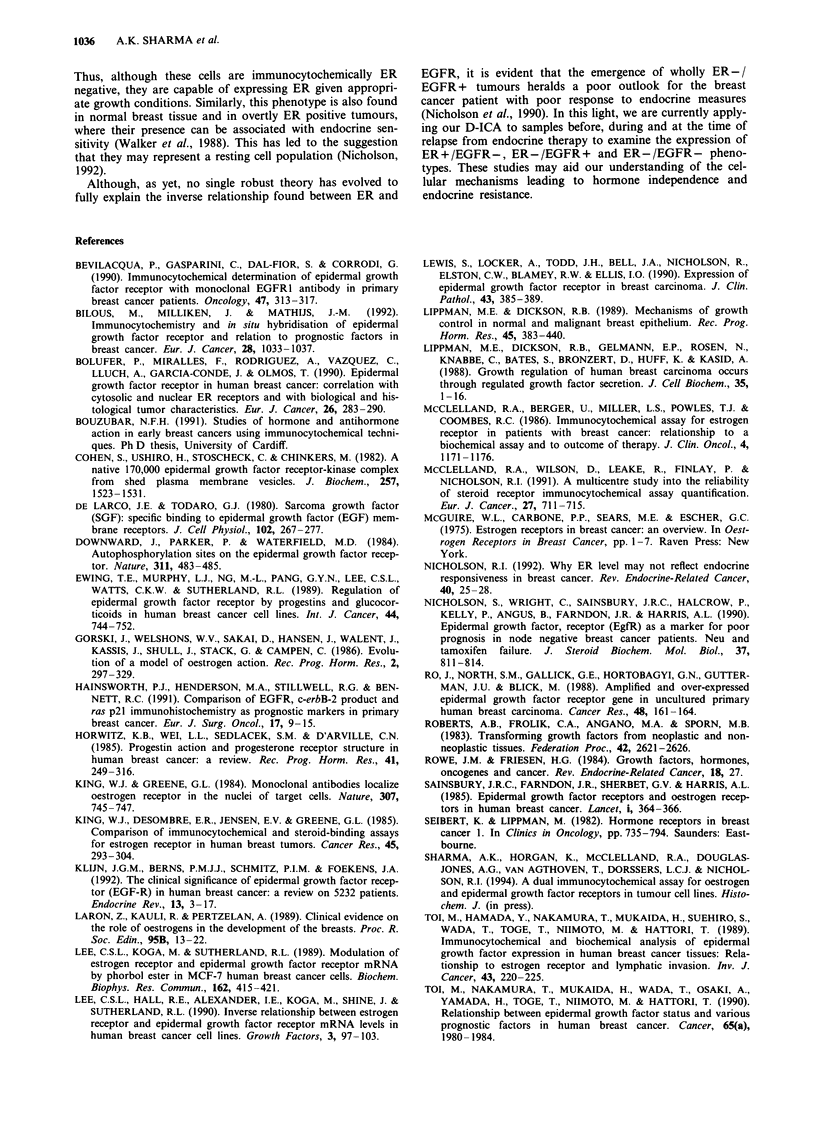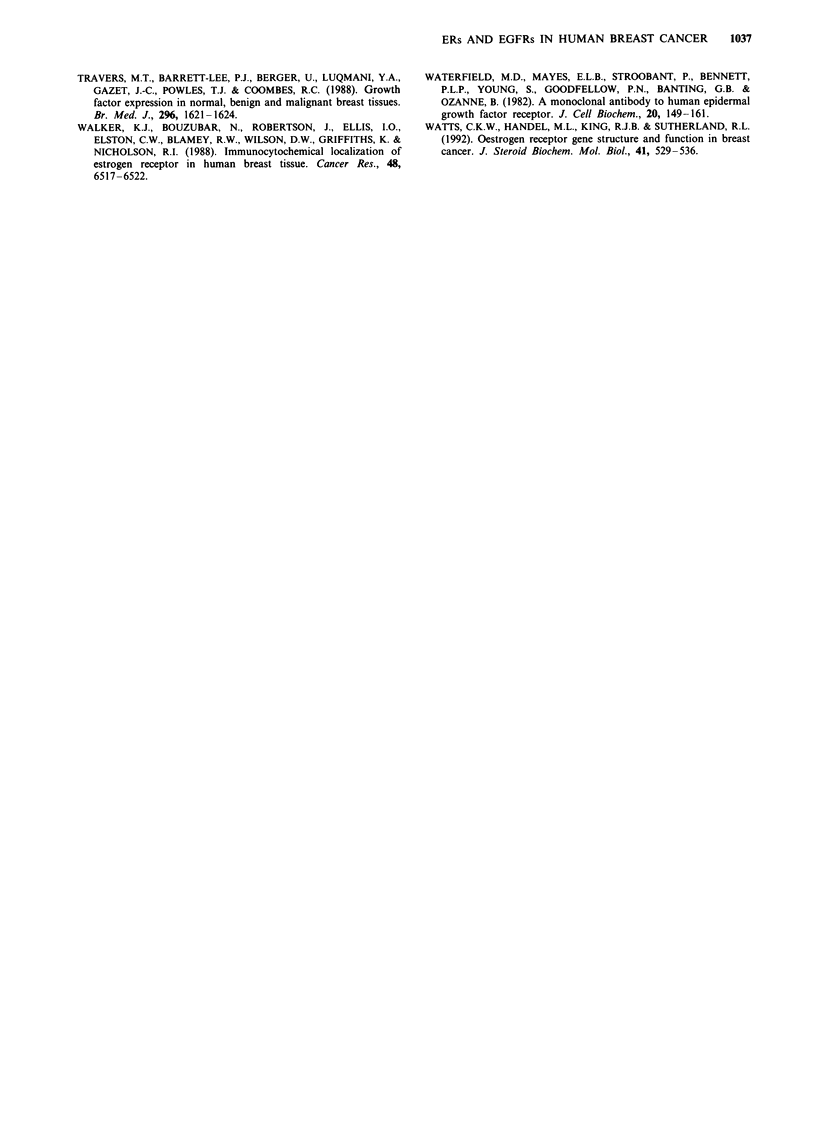# Dual immunocytochemical analysis of oestrogen and epidermal growth factor receptors in human breast cancer.

**DOI:** 10.1038/bjc.1994.203

**Published:** 1994-06

**Authors:** A. K. Sharma, K. Horgan, A. Douglas-Jones, R. McClelland, J. Gee, R. Nicholson

**Affiliations:** Department of Surgery, University of Wales College of Medicine, Heath Park, Cardiff, UK.

## Abstract

**Images:**


Br. J. Cancer (1994), 69, 1032-1037 © Macmillan Press Ltd., 1994

### Dual immunocytochemical analysis of oestrogen and epidermal growth factor receptors in human breast cancer

A.K. Sharma[1], K. Horgan[1], A. Douglas-Jones[2], R. McClelland[3], J. Gee[3] & R. Nicholson[3]

*Departments of [1]Surgery, [2]Pathology and [3]Tenovus Cancer Research Centre, University of Wales College of Medicine, Heath Park, Cardiff, CF4 4XN, UK.*

**Summary** Recent studies have demonstrated a consistent inverse relationship between oestrogen receptor (ER) and epidermal growth factor receptor (EGFR) levels in female human breast cancer. Serial cross-section studies have suggested that separate populations of ER+/EGFR− and ER−/EGFR+ cancer cells exist in tumours deemed by immunocytochemical assay (ICA) to be positive for both. We have developed a dual ICA that is able to stain for both ER and EGFR on a single 5 μm frozen section sample of breast tissue. Twenty-two samples of female human breast cancer tissue that exhibited positivity for ER and EGFR by ER-ICA using the H222 monoclonal antibody and EGFR-ICA using the EGFR1 monoclonal antibody underwent the dual ICA. There was a significant correlation in receptor positivity between the single and dual assays for both ER ($r_s = 0.801$, $P<0.001$) and EGFR ($r_s = 0.831$, $P<0.001$). Individual cancer cells exhibited one of three staining patterns: nuclear staining only (ER+/EGFR−), membrane-associated and cytoplasmic staining only (ER−/EGFR+) or no staining (ER−/EGFR−). No cancer cells exhibited both nuclear and membrane/cytoplasmic staining. This is the first description of a simultaneous dual immunocytochemical assay system for ER and EGFR in clinical breast cancer specimens. The results suggest that ER and EGFR expression are mutually exclusive within an individual breast cancer cell *in vivo* with separate populations of ER+/EGFR− cells, ER−/EGFR+ cells and ER−/EGFR− cells coexisting.

Oestrogens are essential factors in the growth and development of normal breast tissue (Laron *et al.*, 1989) and act via specific nuclear receptors (ERs) (Gorski *et al.*, 1986). In the normal breast oestrogens act as mitogens and also induce the progesterone receptor, which is required for development and differentiation (Horwitz *et al.*, 1985). However, it has long been known that oestrogens have an important role in the development and subsequent progression of human breast cancer (Seibert & Lippman, 1982). More recently evidence has emerged suggesting that oestrogenic effects are mediated to some degree by peptide growth factors and their receptors (Lippman & Dickson, 1989).

These peptide growth factors along with their receptors are now being increasingly recognised for the role that they play in the growth and differentiation of both normal (Rowe & Friesen, 1984) and malignant tissue (Lippman *et al.*, 1988). Of these, the epidermal growth factor receptor (EGFR) is of particular interest in human breast cancer. Human EGFR is a high-affinity transmembrane receptor glycoprotein with a molecular weight of 170,000 (Cohen *et al.*, 1982). It consists of an external ligand-binding domain, a transmembrane section and a cytoplasmic or internal domain that contains a tyrosine-specific protein kinase (Downward *et al.*, 1984).

Many studies in recent years have shown an inverse relationship between ER and EGFR in female human breast cancer (Sainsbury *et al.*, 1985; Toi *et al.*, 1989; Lewis *et al.*, 1990). EGFR expression in human breast cancer is associated with a number of features of poor prognosis, including high tumour grade (Hainsworth *et al.*, 1991), lymphatic invasion (Toi *et al.*, 1989, 1990), lymph node involvement (Hainsworth *et al.*, 1991) and markers of increased cellular proliferation such as Ki-67 (Toi *et al.*, 1990). It has also been shown to be significantly associated with resistance to hormone therapy (Nicholson *et al.*, 1990), reduced relapse-free period (Lewis *et al.*, 1990; Nicholson *et al.*, 1990) and reduced overall survival (Nicholson *et al.*, 1990). There are some studies that dispute these findings, and these have been extensively reviewed elsewhere (Klijn *et al.*, 1992).

This inverse relationship with all its implications for prognosis has been the subject of intense study. Initial investigations involved the use of biochemical ligand-binding assays and revealed that ER could be detected in 60-80% of human breast cancers (McGuire *et al.*, 1975) and EGFR in 45% (Klijn *et al.*, 1992). These studies involved the homogenisation of sample tissue and thus were unable to address the issues of tumour heterogeneity, such as which cells were receptor positive (e.g. benign or malignant) or what proportion of cancer cells were receptor positive. The development of monoclonal antibodies and immunocytochemical assays specific for ER (King & Greene, 1984) and EGFR (Waterfield *et al.*, 1982) on frozen sections addressed these issues. The results of the ER immunocytochemical assay (ER-ICA) using the monoclonal antibody H222 shows high concordance with the biochemical ligand-binding assays (McClelland *et al.*, 1986). ER is immunolocalised in the nuclei of target cells (King & Green, 1984; King *et al.*, 1985), and human breast cancer is found to have a heterogeneous population of ER-positive cells and ER-negative cells (King *et al.*, 1985). The EGFR immunocytochemical assay (EGFR-ICA) using the monoclonal antibody EGFR1 (Waterfield *et al.*, 1982) localises EGFR on the cellular membrane and this also displays a heterogeneous distribution (Toi *et al.*, 1989).

Studies have tried to examine the heterogeneous distribution of ER and EGFR at the individual cellular level by examining serial cross-sections of frozen breast cancer (Toi *et al.*, 1989). The results suggested that EGFR was selectively stained on ER-negative cells and cell groups in tumours categorised to be positive for both. However, the technique of taking serial cross-section samples is open to the criticism that the same cell or cell groups are not being examined in different sections. To address this issue we have developed a dual immunocytochemical assay (D-ICA) that is able to stain for both ER and EGFR on a single 5 μm frozen section sample of tumour cell lines (Sharma *et al.*, 1994). This assay was able to detect all four phenotypes with respect to ER and EGFR (ER+/EGFR−, ER−/EGFR+, ER−/EGFR− and ER+/EGFR+). In the present study we have modified this assay to examine female human breast cancers that have been categorised to be positive for both receptors by ER-ICA and EGFR-ICA.

Correspondence: A.K. Sharma.
Received 8 June 1993; and in revised form 19 January 1994.

### Materials and methods

### *Materials*

The tissues used for analysis were obtained from 22 patients who had previously been categorised to be positive for ER and EGFR by immunocytochemical assay. Of the 22 patients (age range 35–81 years), nine were premenopausal and 13 were post-menopausal (Table I). All the samples were obtained from primary breast cancer patients undergoing mastectomy or wide local excision or from excision of locoregional recurrences in patients who had not received any form of adjuvant therapy.

The samples were snap frozen within 30 min of excision in liquid nitrogen or dry ice. They were then placed in individual flexible plastic moulds that contained a semiviscous freezing compound (OCT, Miles Laboratories, Naperville, IL, USA), snap frozen to −70°C in dry ice and stored at −70°C. Five micron cryostat sections were cut and thaw mounted onto poly-L-lysine tissue adhesive-coated glass slides and immediately either underwent the fixation procedure for the ER-ICA, EGFR-ICA and D-ICA or were stained with haematoxylin–eosin for histological examination. All 22 samples were deemed histologically assessable on the haematoxylin–eosin preparation. Repeat ER-ICA and EGFR-ICA and the D-ICA were performed on all 22 samples.

### *ER-ICA*

The cryostat sections were fixed in 3.7% formaldehyde–PBS (0.01 M phosphate-buffered saline) for 15 min, washed twice for 5 min in PBS, fixed at −10°C in absolute methanol for 6 min followed by immersion in acetone at −10°C for 3 min then washed again in PBS (2 × 5 min). The sections were then stored −20°C in a glycerol–sucrose specimen storage medium for up to 7 days prior to staining. The reagents used for staining were obtained in kit form (ER-ICA monoclonal, Abbott Laboratories, North Chicago, IL, USA). After blocking non-specific binding with normal goat serum (NGS, 15 min), the staining procedure involved sequential incubations, with intervening PBS washes (2 × 5 min), with rat monoclonal anti-ER or control normal rat IgG (30 min), bridging goat anti-rat IgG (30 min), rat peroxidase–anti-peroxidase complex (30 min) and diaminobenzidine (DAB)-hydrogen peroxide–chromogen substrate solution (6 min). The indirect peroxidase–anti-peroxidase procedures of the assay are fully described elsewhere (Walker *et al.*, 1988). The slides were then rinsed in deionised water (2 × 5 min), counterstained with 1% aqueous methyl green (5 min) washed again in deionised water (1 min), dehydrated in graded ethanols (50%, 70%, 90%, 100%), air dried for 1 h, cleared in xylene and coverslipped using a xylene-soluble mountant.

Parallel control sections using normal rat IgG antiserum were run to check for non-specific staining. Inclusion of control slides of MCF-7 cells enabled inter-assay variations to be monitored. Brown nuclear staining is observed in ER-positive cells following the ER-ICA.

### *EGFR-ICA*

The cryostat sections were air dried for 1 h prior to storage in a sealed box at −70°C for up to 7 days prior to assay. The slides were rehydrated in PBS followed by fixation in 50:50 chloroform–acetone at 4°C (10 min) followed by washing in PBS (2 × 5 min). The sections were then incubated with 10% normal goat serum (NGS) in PBS to block non-specific antibody binding. Excess serum was removed and mouse monoclonal primary anti-EGFR antibody (EGFR1, Amersham International, UK) or a control mouse anti-sheep erythrocyte antibody (MASE, sera-Lab, UK) was added (60 min). EGFR1 was added at 1 $\mu$g ml$^{-1}$ with 10% NGS and 5% normal human serum (NHS) in PBS. This monoclonal antibody detects the native folded external domain of human EGFR and does not compete with EGF for binding with EGFR. The control antibody was added at a similar concentration to parallel sections of each specimen. Following the primary incubation the slides were washed in PBS (3 × 5 min) followed by incubation with rabbit anti-mouse peroxidase-conjugated antiserum (Dakopatts, UK) at 1:50 dilution in PBS and containing 10% NGS and 5% NHS (30 min). The slides were then washed in PBS (3 × 5 min) and immunoreactivity was revealed by incubation with 3-amino, 9-ethylcarbazole (AEC) containing hydrogen peroxide obtained in kit form (Immustain, DPC, Oxfordshire, UK) for 10 min. The slides were then washed in tap water (2 × 5 min), counterstained with 1% aqueous methyl green (5 min), washed again in deionised water (1 min) and immediately coverslipped using Aquamount (Gurr, Poole, UK).

Inclusion of control slides of an EGFR-positive breast cancer determined by both radioligand binding and immunocytochemical assays enabled inter-assay variations to be monitored. Red membrane-associated and cytoplasmic staining is observed in EGFR-positive cells following the EGFR-ICA.

### *D-ICA*

The cryostat sections were fixed and stored for up to 7 days as previously described (Sharma *et al.*, 1993). The slides then underwent the sequential steps as per the ER-ICA until the incubation with the DAB–hydrogen peroxide chromogen substrate solution, which was applied for 10 min. This extended incubation period achieved maximal colour production and was followed by washing in deionised water (5 min) then PBS (5 min). The sections were then incubated with 20% NGS and 10% NHS in PBS for 30 min to block non-specific antibody binding. Following the removal of excess serum EGFR1 at 2 $\mu$g ml$^{-1}$ with 10% NGS and 5% NHS in PBS was added to the slides that had received the rat monoclonal anti-ER antiserum for 60 min. MASE, at a similar concentration, was added to the slides that had received the control normal rat IgG for 60 min. The EGFR1 had been preincubated with 2.5 volumes of NHS and the MASE with two volumes of NHS for 30 min prior to final dilutions. The sections were then washed in PBS (3 × 5 min) prior to adding the rabbit anti-mouse peroxidase-conjugated antiserum at 1:50 dilution in PBS with 10% NGS and 5% NHS (30 min). This had also been preincubated with 2.5 volumes of NHS for 30 min prior to final dilution. The remaining steps were as per the EGFR-ICA with the exception that the incubation with AEC lasted 30 min.

### *Assessment and scoring*

The sections were examined, using an Olympus BH2 light microscope with a dual-viewing attachment, by two observers (A.S. and R.M.) and scored according to the intensity of staining and proportion of cells stained. For ER, scores 0–3 were allocated by 0 = no staining; 1 = weak nuclear staining; 2 = moderate nuclear staining; and 3 = strong nuclear staining. For EGFR, scores 0–3 were allocated by 0 = no staining; 1 = faint cytoplasmic reactivity; 2, distinct cytoplasmic or weak membrane reactivity and 3 = strong membrane reactivity. A percentage estimation of cancer cells stained was made for each category and the final result was obtained by the summation of all cells that displayed any reactivity (1 + 2 + 3).

Statistical analysis was performed by using Spearman's rank correlation.

### Results

### *ER-ICA and EGFR-ICA*

In agreement with the previous ER-ICA results, brown nuclear ER staining was immunolocalised in all 22 cases. The proportion of ER-ICA-positive cells was highly variable between patients, with positivity ranging from 10% to 90%

(mean = 49%, Figure 1 and Table I). There was marked heterogeneity in staining intensity between individual cancer cells.

Similarly, red membrane-associated and cytoplasmic EGFR staining was also immunolocalised in all 22 cases. Positivity ranged from 2% to 70% (mean = 21%, Figure 2 and Table I). Heterogeneity between individual cancer cells was less apparent than with ER staining. No staining was observed on the control slides in either assay.

### *D-ICA*

Using the dual ER and EGFR assay, the patterns of immunostaining were essentially similar to those previously described using the single-assay procedures. Thus, brown nuclear ER staining was immunolocalised in all 22 cases and displayed a similar heterogeneous pattern to that seen following the ER-ICA (Figure 3), with positivity ranging from 10% to 90% (mean 46.5%, Figure 1 and Table I). Red membrane-associated and cytoplasmic EGFR staining was immunolocalised in 20/22 (90.9%) cases (Figure 4) with positivity ranging from 0% (two cases) to 80% (mean = 19.5%, Figure 2 and Table I). There was a significant correlation between the single and dual assays in determining both the ER content ($r_s = 0.801$, $P < 0.001$) and the EGFR content ($r_s = 0.831$, $P < 0.001$). There was no staining on the control slides.

**Figure 1** ER levels in the single and dual immunocytochemical assays are significantly correlated ($r_s = 0.801$, $P < 0.001$). The bars represent mean receptor positivity.

**Table I** Patient data and results

| Case | Age (years) | Menopausal status | ER (%) | EGFR (%) | Dual assay (%) ER | EGFR |
|---|---|---|---|---|---|---|
| 1 | 35 | Pre | 50 | 30 | 50 | 20 |
| 2 | 36 | Pre | 65 | 30 | 60 | 30 |
| 3 | 40 | Pre | 30 | 10 | 30 | 5 |
| 4 | 43 | Pre | 50 | 30 | 30 | 20 |
| 5 | 43 | Pre | 40 | 10 | 70 | 10 |
| 6 | 43 | Pre | 40 | 10 | 30 | 10 |
| 7 | 45 | Pre | 60 | 5 | 70 | 5 |
| 8 | 45 | Pre | 20 | 5 | 30 | 10 |
| 9 | 48 | Pre | 10 | 10 | 20 | 0 |
| 10 | 52 | Post | 30 | 60 | 20 | 30 |
| 11 | 58 | Post | 50 | 10 | 50 | 5 |
| 12 | 59 | Post | 90 | 5 | 90 | 5 |
| 13 | 59 | Post | 50 | 10 | 50 | 40 |
| 14 | 61 | Post | 50 | 10 | 50 | 10 |
| 15 | 61 | Post | 30 | 70 | 20 | 80 |
| 16 | 62 | Post | 60 | 5 | 60 | 5 |
| 17 | 63 | Post | 60 | 40 | 40 | 50 |
| 18 | 65 | Post | 50 | 20 | 50 | 20 |
| 19 | 67 | Post | 50 | 30 | 50 | 20 |
| 20 | 72 | Post | 80 | 20 | 80 | 20 |
| 21 | 74 | Post | 50 | 40 | 50 | 30 |
| 22 | 81 | Post | 60 | 2 | 60 | 0 |

Three staining patterns were seen in our samples in the D-ICA.

1. Brown nuclear staining with no membrane/cytoplasmic staining (ER+/EGFR− cells).
2. Red membrane/cytoplasmic staining with no nuclear staining (ER−/EGFR+ cells).
3. No nuclear or membrane/cytoplasmic staining (ER−/EGFR− cells).

No cells displaying both nuclear and membrane/cytoplasmic staining were identified (Figure 5). Summation of the proportion of cells expressing either ER or EGFR never exceeded 100% in either the single or dual assays for any individual patient (Table I).

In this small select group of double-positive patients we found no significant inverse linear relationship between ER and EGFR in either the single assays (Figure 6, $r_s = -0.163$) or the dual assay (Figure 7, $r_s = -0.249$). With the exception of two patients EGFR positivity did not exceed 40%.

### Discussion

The present study is the first to describe a dual immunocytochemical assay that will stain for both ER and EGFR on single 5 µm frozen section samples of clinical human breast cancer specimens. The results of staining for ER and EGFR following the D-ICA are similar to those achieved in the two single assays of proven prognostic significance (McClelland *et al.*, 1986; Lewis *et al.*, 1990) with only small variations in the proportion of tumour cells being detected as positive. Such relatively minor variations may result from observer errors, which are inherent in immunocytochemical procedures (McClelland *et al.*, 1991), or may be due to tumour heterogeneity, which is well documented (Walker *et al.*, 1988).

Using the dual assay, three phenotypes have been observed in our series of 22 ER-positive/EGFR-positive samples; ER+/EGFR− cells, ER−/EGFR+ cells and ER−/EGFR− cells. Importantly, no ER+/EGFR+ cells were identified, despite the proven ability of this assay to detect such cells when they have been artificially constructed by transfection procedures (Sharma *et al.*, 1994). These data suggest that ER and EGFR are mutually exclusive within an individual cancer cell *in vivo*, a conclusion that is supported by studies examining the expression of these receptors in serial cross-section analysis (Toi *et al.*, 1989). An alternative explanation is that ER+/EGFR+ cells were not detected because of the relatively small number of cases examined. However, it is difficult to accrue large numbers of double-positive tumours as these represent between only 4% (Bilous *et al.*, 1992) to

**Figure 2** EGFR levels in the single and dual assays are also significantly correlated ($r_s = 0.831$, $P < 0.001$). Mean receptor positivity is represented by the bars.

**Figure 3** ER-positive/EGFR-negative cells in the D-ICA displaying brown nuclear staining without any membrane-associated or cytoplasmic staining (original magnification × 990).

**Figure 4** ER-negative/EGFR-positive cells in the D-ICA displaying red membrane-associated and cytoplasmic staining alone without any nuclear staining (original magnification × 990).

**Figure 5** Photograph demonstrating separate populations of ER-positive/EGFR-negative cells **a**, and ER-negative/EGFR-positive cells **b**, coexisting within an individual human breast cancer following the D-ICA. No ER-positive/EGFR-positive cells were observed in the specimens examined (original magnification × 990).

**Figure 6** Relationship between ER and EGFR in the single assays.

**Figure 7** Relationship between ER and EGFR in the D-ICA.

37% (Bevilacqua *et al.*, 1990) of primary breast cancers using immunocytochemical analysis.

Although the exact mechanisms responsible for final expression of ER or EGFR are not fully understood, investigations in clinical breast cancer specimens have shown gene amplification with or without rearrangement to be a relatively rare event (Ro *et al.*, 1988; Watts *et al.*, 1992) and is thus unlikely to significantly contribute to an altered phenotype. In contrast to this, *in vitro* studies on human breast cancer cell lines, while confirming the inverse relationship between ER and EGFR mRNA and protein expression (Lee *et al.*, 1990), have also shown that treatment of ER-positive cell lines by progestins (Ewing *et al.*, 1989) and 12-*O*-tetradecanoyl phorbol 13-acetate (Lee *et al.*, 1989) increases EGFR binding and mRNA levels with concomitant decline in ER binding and mRNA. These data suggest that final receptor expression may be determined by direct regulation of one receptor gene by the product of the other and/or reciprocal control by a common regulator that has opposite effects on these receptors at the transcriptional or post-transcriptional level. The degree to which these findings can be applied to the *in vivo* situation is unclear, although it has been shown that EGFR mRNA is found more commonly in ER-negative than in ER-positive breast cancer biopsies (Travers *et al.*, 1988).

Significantly, it is now well established that oestrogen binding to ER initiates the transcription of various mRNAs, including transforming growth factor α (TGF-α), a ligand for EGFR (Roberts *et al.*, 1983). Since TGF-α/EGFR binding causes down-regulation of EGFR by means of receptor internalisation (De Larco & Todaro, 1980), the induction of TGF-α in ER-positive tumours would tend to suppress EGFR levels. Certainly, EGFR content is higher in the ER-negative tumours (Bolufer *et al.*, 1990), and the present study has shown that in 20/22 cases (90.9%) EGFR expression was 40% or less in these double-positive tumours.

In addition to the ER+/EGFR− and ER−/EGFR+ phenotypes, the current study has also identified ER−/EGFR− cells. Such cells were also observed in our *in vitro* studies (Sharma *et al.*, 1993) and may be a consequence of ER being a cell cycle-related product (Bouzubar, 1991). These cell culture studies have shown that MCF-7 cells in $G_0$ are frequently ER negative and are only induced to express ER as they progress through the cell cycle (Bouzubar, 1991).

Thus, although these cells are immunocytochemically ER negative, they are capable of expressing ER given appropriate growth conditions. Similarly, this phenotype is also found in normal breast tissue and in overtly ER positive tumours, where their presence can be associated with endocrine sensitivity (Walker *et al*., 1988). This has led to the suggestion that they may represent a resting cell population (Nicholson, 1992).

Although, as yet, no single robust theory has evolved to fully explain the inverse relationship found between ER and EGFR, it is evident that the emergence of wholly ER−/EGFR+ tumours heralds a poor outlook for the breast cancer patient with poor response to endocrine measures (Nicholson *et al*., 1990). In this light, we are currently applying our D-ICA to samples before, during and at the time of relapse from endocrine therapy to examine the expression of ER+/EGFR−, ER−/EGFR+ and ER−/EGFR− phenotypes. These studies may aid our understanding of the cellular mechanisms leading to hormone independence and endocrine resistance.

### References

BEVILACQUA, P., GASPARINI, C., DAL-FIOR, S. & CORRODI, G. (1990). Immunocytochemical determination of epidermal growth factor receptor with monoclonal EGFR1 antibody in primary breast cancer patients. *Oncology*, **47**, 313–317.

BILOUS, M., MILLIKEN, J. & MATHIJS, J.-M. (1992). Immunocytochemistry and *in situ* hybridisation of epidermal growth factor receptor and relation to prognostic factors in breast cancer. *Eur. J. Cancer*, **28**, 1033–1037.

BOLUFER, P., MIRALLES, F., RODRIGUEZ, A., VAZQUEZ, C., LLUCH, A., GARCIA-CONDE, J. & OLMOS, T. (1990). Epidermal growth factor receptor in human breast cancer: correlation with cytosolic and nuclear ER receptors and with biological and histological tumor characteristics. *Eur. J. Cancer*, **26**, 283–290.

BOUZUBAR, N.F.H. (1991). Studies of hormone and antihormone action in early breast cancers using immunocytochemical techniques. Ph D thesis, University of Cardiff.

COHEN, S., USHIRO, H., STOSCHECK, C. & CHINKERS, M. (1982). A native 170,000 epidermal growth factor receptor-kinase complex from shed plasma membrane vesicles. *J. Biochem.*, **257**, 1523–1531.

DE LARCO, J.E. & TODARO, G.J. (1980). Sarcoma growth factor (SGF): specific binding to epidermal growth factor (EGF) membrane receptors. *J. Cell Physiol.*, **102**, 267–277.

DOWNWARD, J., PARKER, P. & WATERFIELD, M.D. (1984). Autophosphorylation sites on the epidermal growth factor receptor. *Nature*, **311**, 483–485.

EWING, T.E., MURPHY, L.J., NG, M.-L., PANG, G.Y.N., LEE, C.S.L., WATTS, C.K.W. & SUTHERLAND, R.L. (1989). Regulation of epidermal growth factor receptor by progestins and glucocorticoids in human breast cancer cell lines. *Int. J. Cancer*, **44**, 744–752.

GORSKI, J., WELSHONS, W.V., SAKAI, D., HANSEN, J., WALENT, J., KASSIS, J., SHULL, J., STACK, G. & CAMPEN, C. (1986). Evolution of a model of oestrogen action. *Rec. Prog. Horm. Res.*, **2**, 297–329.

HAINSWORTH, P.J., HENDERSON, M.A., STILLWELL, R.G. & BENNETT, R.C. (1991). Comparison of EGFR, c-*erb*B-2 product and *ras* p21 immunohistochemistry as prognostic markers in primary breast cancer. *Eur. J. Surg. Oncol.*, **17**, 9–15.

HORWITZ, K.B., WEI, L.L., SEDLACEK, S.M. & D'ARVILLE, C.N. (1985). Progestin action and progesterone receptor structure in human breast cancer: a review. *Rec. Prog. Horm. Res.*, **41**, 249–316.

KING, W.J. & GREENE, G.L. (1984). Monoclonal antibodies localize oestrogen receptor in the nuclei of target cells. *Nature*, **307**, 745–747.

KING, W.J., DESOMBRE, E.R., JENSEN, E.V. & GREENE, G.L. (1985). Comparison of immunocytochemical and steroid-binding assays for estrogen receptor in human breast tumors. *Cancer Res.*, **45**, 293–304.

KLIJN, J.G.M., BERNS, P.M.J.J., SCHMITZ, P.I.M. & FOEKENS, J.A. (1992). The clinical significance of epidermal growth factor receptor (EGF-R) in human breast cancer: a review on 5232 patients. *Endocrine Rev.*, **13**, 3–17.

LARON, Z., KAULI, R. & PERTZELAN, A. (1989). Clinical evidence on the role of oestrogens in the development of the breasts. *Proc. R. Soc. Edin.*, **95B**, 13–22.

LEE, C.S.L., KOGA, M. & SUTHERLAND, R.L. (1989). Modulation of estrogen receptor and epidermal growth factor receptor mRNA by phorbol ester in MCF-7 human breast cancer cells. *Biochem. Biophys. Res. Commun.*, **162**, 415–421.

LEE, C.S.L., HALL, R.E., ALEXANDER, I.E., KOGA, M., SHINE, J. & SUTHERLAND, R.L. (1990). Inverse relationship between estrogen receptor and epidermal growth factor receptor mRNA levels in human breast cancer cell lines. *Growth Factors*, **3**, 97–103.

LEWIS, S., LOCKER, A., TODD, J.H., BELL, J.A., NICHOLSON, R., ELSTON, C.W., BLAMEY, R.W. & ELLIS, I.O. (1990). Expression of epidermal growth factor receptor in breast carcinoma. *J. Clin. Pathol.*, **43**, 385–389.

LIPPMAN, M.E. & DICKSON, R.B. (1989). Mechanisms of growth control in normal and malignant breast epithelium. *Rec. Prog. Horm. Res.*, **45**, 383–440.

LIPPMAN, M.E., DICKSON, R.B., GELMANN, E.P., ROSEN, N., KNABBE, C., BATES, S., BRONZERT, D., HUFF, K. & KASID, A. (1988). Growth regulation of human breast carcinoma occurs through regulated growth factor secretion. *J. Cell Biochem.*, **35**, 1–16.

MCCLELLAND, R.A., BERGER, U., MILLER, L.S., POWLES, T.J. & COOMBES, R.C. (1986). Immunocytochemical assay for estrogen receptor in patients with breast cancer: relationship to a biochemical assay and to outcome of therapy. *J. Clin. Oncol.*, **4**, 1171–1176.

MCCLELLAND, R.A., WILSON, D., LEAKE, R., FINLAY, P. & NICHOLSON, R.I. (1991). A multicentre study into the reliability of steroid receptor immunocytochemical assay quantification. *Eur. J. Cancer.*, **27**, 711–715.

MCGUIRE, W.L., CARBONE, P.P., SEARS, M.E. & ESCHER, G.C. (1975). Estrogen receptors in breast cancer: an overview. In *Oestrogen Receptors in Breast Cancer*, pp. 1–7. Raven Press: New York.

NICHOLSON, R.I. (1992). Why ER level may not reflect endocrine responsiveness in breast cancer. *Rev. Endocrine-Related Cancer*, **40**, 25–28.

NICHOLSON, S., WRIGHT, C., SAINSBURY, J.R.C., HALCROW, P., KELLY, P., ANGUS, B., FARNDON, J.R. & HARRIS, A.L. (1990). Epidermal growth factor, receptor (EgfR) as a marker for poor prognosis in node negative breast cancer patients. Neu and tamoxifen failure. *J. Steroid Biochem. Mol. Biol.*, **37**, 811–814.

RO, J., NORTH, S.M., GALLICK, G.E., HORTOBAGYI, G.N., GUTTERMAN, J.U. & BLICK, M. (1988). Amplified and over-expressed epidermal growth factor receptor gene in uncultured primary human breast carcinoma. *Cancer Res.*, **48**, 161–164.

ROBERTS, A.B., FROLIK, C.A., ANGANO, M.A. & SPORN, M.B. (1983). Transforming growth factors from neoplastic and non-neoplastic tissues. *Federation Proc.*, **42**, 2621–2626.

ROWE, J.M. & FRIESEN, H.G. (1984). Growth factors, hormones, oncogenes and cancer. *Rev. Endocrine-Related Cancer*, **18**, 27.

SAINSBURY, J.R.C., FARNDON, J.R., SHERBET, G.V. & HARRIS, A.L. (1985). Epidermal growth factor receptors and oestrogen receptors in human breast cancer. *Lancet*, **i**, 364–366.

SEIBERT, K. & LIPPMAN, M. (1982). Hormone receptors in breast cancer 1. In *Clinics in Oncology*, pp. 735–794. Saunders: Eastbourne.

SHARMA, A.K., HORGAN, K., MCCLELLAND, R.A., DOUGLAS-JONES, A.G., VAN AGTHOVEN, T., DORSSERS, L.C.J. & NICHOLSON, R.I. (1994). A dual immunocytochemical assay for oestrogen and epidermal growth factor receptors in tumour cell lines. *Histochem. J.* (in press).

TOI, M., HAMADA, Y., NAKAMURA, T., MUKAIDA, H., SUEHIRO, S., WADA, T., TOGE, T., NIIMOTO, M. & HATTORI, T. (1989). Immunocytochemical and biochemical analysis of epidermal growth factor expression in human breast cancer tissues: Relationship to estrogen receptor and lymphatic invasion. *Inv. J. Cancer*, **43**, 220–225.

TOI, M., NAKAMURA, T., MUKAIDA, H., WADA, T., OSAKI, A., YAMADA, H., TOGE, T., NIIMOTO, M. & HATTORI, T. (1990). Relationship between epidermal growth factor status and various prognostic factors in human breast cancer. *Cancer*, **65(a)**, 1980–1984.

TRAVERS, M.T., BARRETT-LEE, P.J., BERGER, U., LUQMANI, Y.A., GAZET, J.-C., POWLES, T.J. & COOMBES, R.C. (1988). Growth factor expression in normal, benign and malignant breast tissues. *Br. Med. J.*, **296**, 1621–1624.

WALKER, K.J., BOUZUBAR, N., ROBERTSON, J., ELLIS, I.O., ELSTON, C.W., BLAMEY, R.W., WILSON, D.W., GRIFFITHS, K. & NICHOLSON, R.I. (1988). Immunocytochemical localization of estrogen receptor in human breast tissue. *Cancer Res.*, **48**, 6517–6522.

WATERFIELD, M.D., MAYES, E.L.B., STROOBANT, P., BENNETT, P.L.P., YOUNG, S., GOODFELLOW, P.N., BANTING, G.B. & OZANNE, B. (1982). A monoclonal antibody to human epidermal growth factor receptor. *J. Cell Biochem.*, **20**, 149–161.

WATTS, C.K.W., HANDEL, M.L., KING, R.J.B. & SUTHERLAND, R.L. (1992). Oestrogen receptor gene structure and function in breast cancer. *J. Steroid Biochem. Mol. Biol.*, **41**, 529–536.